# Computational Screening and Experimental Evaluation of Wheat Proteases for Use in the Enzymatic Therapy of Gluten-Related Disorders

**DOI:** 10.3390/ph18040592

**Published:** 2025-04-18

**Authors:** Lyudmila V. Savvateeva, Olga E. Chepikova, Alena D. Solonkina, Artemiy A. Sakharov, Neonila V. Gorokhovets, Andrey V. Golovin, Andrey A. Zamyatnin

**Affiliations:** 1 Institute of Translational Medicine and Biotechnology, Sechenov First Moscow State Medical University, 119991 Moscow, Russia; ludmilaslv@yandex.ru (L.V.S.); chepikovaolga@gmail.com (O.E.C.); gorokhovets@gmail.com (N.V.G.); 2 Faculty of Bioengineering and Bioinformatics, Lomonosov Moscow State University, 119234 Moscow, Russia; alene.s@mail.ru (A.D.S.); golovin.andrey@gmail.com (A.V.G.); 3 Research Center for Translational Medicine, Sirius University of Science and Technology, 354340 Sirius, Krasnodar Region, Russia; ar.sakharov1@gmail.com; 4 Shemyakin-Ovchinnikov Institute of Bioorganic Chemistry, Russian Academy of Sciences, 117997 Moscow, Russia; 5 Belozersky Institute of Physico-Chemical Biology, Lomonosov Moscow State University, 119991 Moscow, Russia; 6 Department of Biological Chemistry, Sechenov First Moscow State Medical University, 119991 Moscow, Russia

**Keywords:** glutenase, gluten, computational screening, celiac disease

## Abstract

**Background:** Gluten-related disorders, particularly celiac disease, are triggered in susceptible individuals by the toxic effects of gluten, the major storage protein of wheat grains. This toxicity can be reduced by wheat glutenases. Members of the papain-like cysteine protease family, which can act in the human gastrointestinal tract, are promising candidates for the enzymatic treatment of celiac disease. **Methods:** Two wheat proteases were selected using AlphaFold2, produced in recombinant forms, and characterized. Their glutenase potentials under acidic or slightly acidic conditions were evaluated and compared with the properties of the previously characterized wheat glutenase Triticain-α. **Results:** All enzymes tested, Ta-P7, Ta-V6, and Triticain-α, were able to hydrolyze the model substrate (α-gliadin-derived epitope) in the pH range of 3.6–7.5. Nevertheless, Triticain-α performs the most efficient hydrolysis of the peptide substrate under the conditions of the gastrointestinal tract, according to its kinetic characteristics. In the wheat gluten degradation experiment at pH 4.6 and 37 °C, both Ta-P7 and Triticain-α cleaved the mixture almost completely within 5 min. In addition, Triticain-α and Ta-P7 significantly reduced the levels of toxic peptides compared to both intact gluten and gluten treated with pepsin-trypsin digestion as tested by the Ridascreen Gliadin Kit. **Conclusions:** Novel wheat proteases under investigation possess the expected glutenase activity to varying degrees; however, Triticain-α is a primary candidate for potential use in the enzymatic therapy of gluten-related disorders.

## 1. Introduction

Wheat (or gluten)-related disorders are diseases caused by ingestion of gluten, specifically celiac disease (CD), non-celiac gluten sensitivity, and wheat allergy [1]. According to recent data, CD has an estimated global prevalence of up to 1% [2]. At the same time, there is evidence that the incidence of CD is decreasing in countries with a high prevalence of CD [3]. Due to increased awareness and diagnostic availability, the number of registered cases of CD is expected to increase worldwide (mainly in Asian countries) in the next few years [4,5,6,7].

CD is caused by gluten, which is ingested with food and causes disease in susceptible individuals. The most important feature of gluten proteins with respect to their role in the development of CD is the presence of water-insoluble protein domains—prolamins (gliadins in wheat), consisting of repetitive sequences with high proline and glutamine content and characterized by high resistance to proteolytic digestion in the gastrointestinal tract by the pancreas and intestine [8,9], resulting in the formation of toxic peptides (mapped immunogenic epitopes) that immunostimulate intestinal HLA-DQ2/DQ8-restricted gluten-specific CD4+ T cells [2].

A strict, lifelong gluten-free diet (GFD) remains the mainstay of treatment for CD, and the availability of gluten-free options is increasing. However, adherence to a strict gluten-free diet is not always possible, mainly due to accidental exposure to gluten and the low quality and high cost of gluten-free products [10]. As a result, many patients are unable (or unwilling) to adhere to the diet, which can lead to complications in patients with gluten intolerance. Therefore, the development of alternative innovative approaches to gluten neutralization, both at the production level in the food industry (e.g., the development of new alternative flour products) and in the development of drug therapies, is being actively explored [8,10,11].

Gluten is the major protein group that accumulates in grains to support germination and seedling development. Proteolysis of prolamins during germination of wheat (or other grains) is accomplished by the activity of endoproteases, and the resulting oligopeptides are substrates for exopeptidases that sequentially remove amino acids from their amino or carboxyl termini [11]. The proteases responsible for this process are either synthesized and stored in the endosperm during grain development or synthesized and secreted during germination from the aleurone or shield that completely surrounds the endosperm [11]. More specifically, during germination, the endosperm is acidified to pH 5 by organic acids released from the aleurone, creating an optimal pH for proteolysis that promotes the solubilization of prolamins. Initial cleavage is accomplished by cysteine endoproteases secreted into the endosperm by the aleurone, which are unique to cereals and are involved in protein degradation during programmed cell death [11,12,13]. Therefore, it is reasonable to hypothesize that proteases present in germinating grains, and in particular wheat proteases (or, as a variant, proteases of cereal insect pests or microbial pests that infest food grains), represent perspective candidates for their use in food processing or therapeutics to degrade immunotoxic gluten protein in an appropriate environment. Following this proposal, a number of studies have been carried out on plant enzymes for the degradation of cereal storage proteins [14,15,16,17,18]. Particular attention was paid to the proteases resistant to acidic pH and to digestive enzymes present in the human stomach, making them potential drug candidates for enzymatic therapy assays. However, due to a number of limitations resulting in low clinical efficacy, none of these drug candidates are currently approved for clinical use [19,20,21].

The goal of our study is to identify a potential wheat glutenase that is self-activating, most active toward and resistant to acidic pH, efficient at degrading gluten, and particularly efficient at degrading toxic gluten-derived peptides. To achieve this goal, we used both bioinformatics tools and biochemical assays. Computational screening of all 181 wheat-encoded papain-like cysteine proteases (PLCPs) [22], most of which are active at acidic pH as members of the C1A family, was used to identify their substrate specificity for peptides containing gluten-specific motifs, followed by biochemical characterization and comparison of their gluten-degrading properties with the previously characterized wheat glutenase Triticain-α [23]. The results will help to select the most promising protease candidates for further clinical applications in celiac disease.

## 2. Results

**Figure 1:**
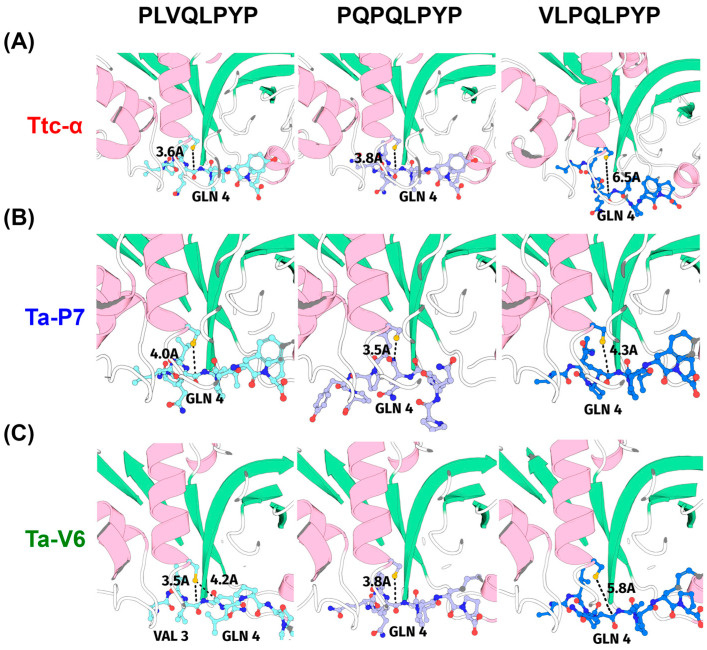
Enzyme substrate model for the wheat proteins of *Triticum aestivum* L. The enzyme is shown as a cartoon, the substrate as balls and sticks, the labels GLN4 (Q4) and VAL3 (V3) annotate cleavage sites in the substrate, and the distance from the attacking sulfur atom to the attacked carbonyl is shown as a black dotted line. Representation of the complex Ttc-α (**A**), Ta-P7 (**B**), or Ta-V6 (**C**) with the peptides PLVQLPYP (**left** panel), PQPQLPYP (**middle** panel), and VLPQLPYP (**right** panel).

Optimal enzyme search with AlphaFold2-multimer-v3 (AFm) screening was performed to determine the substrate specificity of wheat-encoded 181 PLCPs [22] with respect to peptides containing repetitive gluten-specific motifs, namely PLVQLPYP, PQPQLPYP, and VLPQLPYP [23]. AFm was used to build an enzyme-substrate complex with extended sampling using eight seed numbers and five models from amber geometry optimization; this step resulted in 6240 relaxed structures. The position of the catalytic cysteine residue was not known in advance, so the distances from the known carbonyl carbon in the cleavage site to all sulfur atoms in the cysteines were measured, and the closest was used to sort the results by distance magnitude. This trivial step resulted in some cases where the position of the peptide did not correspond to the catalytic site. Our initial hypothesis was that selectable wheat proteases should work equally well with PLVQLPYP, PQPQLPYP, and VLPQLPYP (Figure 1).

The results were sorted by the average distance from the catalytic cysteine to the attacked carbonyl (Table S1), and the best scores for the indicated peptides corresponded to the putative protein identified as TRIAE_AA1165320.1 (or UNIPROT id A0A3B6JDP7), hereafter referred to as Ta-P7 (Figure 1B). It is interesting to note that Triticain-α (Ttc-α), with the identifier TRIAE_AA0430650.1 (Figure 1A), was ranked 50th in the sorted list (Table S1). Upon visual inspection of the highest quality structural models, we observed that in some instances within the model of the complex PLVQPYP with TRIAE_AA0280660.1 (or UNIPROT id A0A3B6B7V6, hereafter referred to as Ta-V6), the peptide exhibited a positional shift of one residue (Figure 1C). This shift resulted in potential cleavage occurring at valine (VAL3, V3) rather than glutamine (GLN4, Q4), relative to the optimal binding conformation observed in other complexes with this substrate (Figure 1C). Therefore, two novel protein sequences, Ta-P7 and Ta-V6, and Triticain-α as a comparative control, were selected for further experimental evaluation.

**Figure 2:**
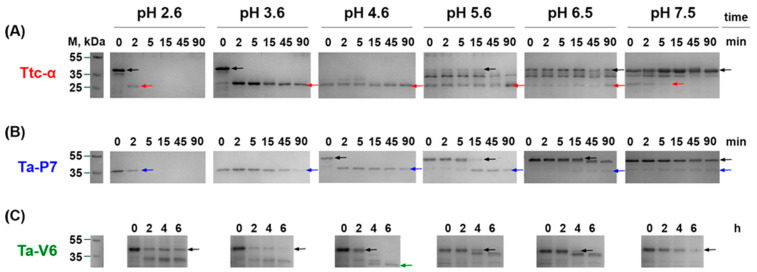
SDS-PAGE analysis of the wheat proteases Ttc-α (**A**), Ta-P7 (**B**), and Ta-V6 (**C**) autocatalytically activated at 37 °C in buffers of different pH. Masses in kDa are given in the left column; arrows indicate enzyme forms (black arrows indicate the proenzyme of each protease; red, blue, or green arrows indicate the mature form of Triticain-α, Ta-P7, and Ta-V6, respectively).

The proteins Ta-P7 and Ta-V6 were expressed as proenzymes in *Escherichia coli* in the soluble form and purified by affinity and size exclusion chromatography. Incubation of the inactive proenzymes in the pH range of 2.6 to 7.5 at 37 °C led to the formation of active forms of these proteases in a similar manner (Figure 2). Thus, the detection of mature Ta-P7 of approximately 36 kDa was observed almost immediately at pH 2.6–3.6 and after 5 min of incubation at pH 4.6, with longer incubation times required at higher pH values to obtain active enzyme (Figure 2B). In the case of Ta-V6, however, a mature enzyme of approximately 28 kDa was only achieved at pH 4.6 after 6 h of incubation (Figure 2C). The present results confirmed that Triticain-α is autocatalytically activated with a molecular weight of the active proteolytic domain of approximately 32 kDa [23] under the study conditions (Figure 2A). The results based on these data showed that the studied proteases exhibited optimal autocatalytic activation under acidic conditions (pH 4.6) at 37 °C.

**Figure 3:**
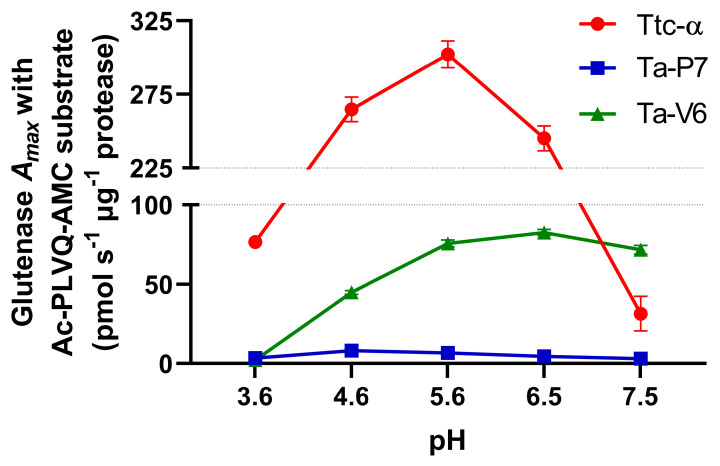
pH dependence of the rate of hydrolysis of Ac-PLVQ-AMC by recombinant Ttc-α, Ta-P7, and Ta-V6 at 37 °C. Error bars indicate the standard error of the mean.

Ta-P7 and Ta-V6, as well as Triticain-α (as a comparative control), were evaluated for enzymatic cleavage specificity using a fluorogenic peptide substrate derived from the gluten protein components, Ac-PLVQ-AMC [23,24], in buffers of different pH. Based on the obtained characteristics of the enzymatic reaction, the kinetic parameters of Ac-PLVQ-AMC hydrolysis, namely the Michaelis constant (*K*_M_), the maximum enzyme activity (*A*_max_), and the catalytic constant (*k*_cat_), were calculated (Table 1 and Figure 3). The results showed that Ac-PLVQ-AMC was most efficiently cleaved by Triticain-α, especially at pH 3.6–5.6. Ta-P7 hydrolyzed Ac-PLVQ-AMC inefficiently over the entire pH range studied, despite comparable affinity for the substrate. In contrast, Ta-V6 cleaved Ac-PLVQ-AMC less efficiently than Triticain-α, with increasing efficiency with increasing pH (Table 1 and Figure 3).

**Figure 4:**
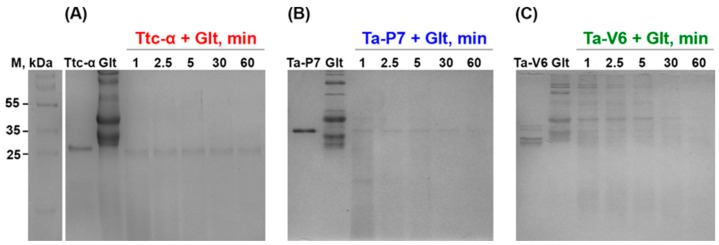
SDS-PAGE analysis of gluten (Glt) degradation by Ttc-α (**A**), Ta-P7 (**B**), and Ta-V6 (**C**) at 37 °C in pH 4.6 during 60 min incubation. The positions of the molecular weight standards (kDa) are shown in the left column.

The degradation of the bulk wheat gluten fraction by the new recombinant Ta-P7 and Ta-V6, as well as Triticain-α (as a comparative control), was first studied by incubating the corresponding reaction mixtures at 37 °C and pH 4.6, followed by analysis by SDS-PAGE. The protein bands that appeared in Coomassie-stained gels in samples treated with the corresponding enzymes were compared to undigested gluten, indicating degradation of the latter. As shown in Figure 4, after 1 h of incubation for all the enzyme mixtures studied with gluten, the bands of the latter underwent almost complete degradation, and for both Ta-P7 and Ttriticain-α, this process took less than 5 min (Figure 4A,B), while Ta-V6 required from 30 to 60 min (Figure 4C).

**Figure 5:**
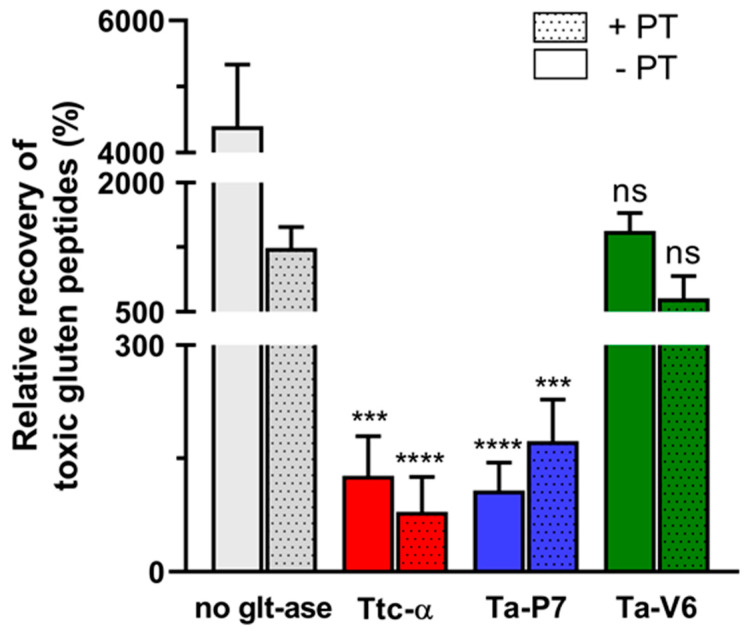
Analysis of gluten fractions and their glutenase digests at 37 °C, pH 4.6 using the Ridascreen^®^ Gliadin competitive ELISA kit. (Glt-ase—tested glutenase: Ta-P7, Ta-V6 or Ttc-α; PT—pepsin-trypsin digest). Error bars indicate the standard error of the mean; ns—*p* > 0.05; ***—*p* = 0.0001; ****—*p* < 0.0001 vs. PT-digested gluten only control.

The Ridascreen^®^ Gliadin competitive ELISA kit was used to determine the amount of gluten-derived toxic peptides degraded during the fermentation of wheat gluten suspension (in 0.2 M glycine buffer, pH 2.6) by the tested proteases Ta-P7, Ta-V6, or Triticain-α with or without pepsin and trypsin. The obtained results were calculated as a value of relative recovery as follows: toxic peptides [ng mL^−1^]/gluten concentration [ng mL^−1^] × mass fraction of gluten [25], followed by presentation as a histogram (Figure 5).

As shown in Figure 5, the gluten fractions in the presence of the studied glutenases gave lower recoveries compared to the PT digest alone, with Triticain-α and Ta-P7 being particularly effective. In addition, it was statistically demonstrated that Triticain-α was effective in reducing the levels of toxic gluten components in the presence of PT digest under the conditions studied. No significant difference was observed in samples treated with Ta-V6.

## 3. Discussion

The principle of using enzymes to treat gluten-dependent disorders is to inactivate or destroy toxic gluten peptides (and other prolamins) before they reach the intestine. There are two main approaches: pre-treatment of foods with enzymes or oral administration of enzymes as a drug or supplement. Such foods would be in demand for inclusion in the diet of people with gluten intolerance. Enzymes that are being studied to reduce the immunoreactivity of toxic peptides include transglutaminases and peptidases of various origins (plant, fungal, bacterial, and animal proteases or their derivatives) [26].

For oral enzyme therapy, it is important that the glutenases used are resistant to gastric pH and human digestive enzymes in order to remain active under gastric digestive conditions. In addition, safety, high specificity, and rapid activation and action under gastrointestinal conditions are prerequisites for the development of promising enzyme therapeutics. In addition, the production of enzymes for therapeutic purposes must be economically viable and reliable. Glutenases that meet these requirements can be used by patients, especially those with celiac disease, both to avoid complications from accidental ingestion of gluten and to enable them to consume not only gluten-free but also low-gluten foods, thereby broadening their diets.

Among the various glutenases potentially suitable for use in oral enzyme therapy, whose properties have been described in some studies (and mentioned below), it is possible to identify only a few that show optimal specific activity in the acidic and slightly acidic environment characteristic of the human intestine and gastrointestinal tract [27]. In particular, fungal enzymes from *Aspergillus niger*, proline-specific endopeptidase (AN-PEP), and aspergillopepsin (ASP), have an optimum at pH 4–5 and pH 3, respectively. AN-PEP has been shown to be highly efficient in degrading CD-active peptides [28], whereas ASP is able to extensively hydrolyze dietary gluten into short peptides but lacks specificity for 33-mer peptides of α2-gliadin [29]. ASPs have already been shown to be safe for human consumption and can be added to more potent and specific glutenases (e.g., EP-B2 or some microbial prolyl endopeptidases) to further enhance their therapeutic potency. A bacterial protease from *Actinoallomurus* sp. *A8*, E40, efficiently degrades the most immunogenic 33-mer as well as whole gliadin proteins, with optimal activity at pH 3–6 [30]. The streptomycetes used to produce recombinant E40 are considered a safe protein source for dietary consumption, making E40 a suitable candidate for dietary treatment of gluten toxicity [30,31]. Another engineered endopeptidase of microbial origin, kumamolisin from *Alicyckobacillus sendaiensis*, Kuma030, has been shown to degrade a broad spectrum of immunogenic gliadin epitopes under gastric conditions (pH 4). The potential of Kuma030 as a therapeutic agent for celiac disease will be determined in further studies [32].

Few plant glutenases have been described that act under acidic conditions. A study on the proteolytic components of carnivorous insectivorous plants (*Nepenthes* spp.) [33] showed that they are promising for use in enzyme therapy due to the action of the enzymes nepenthesin and neprosin. The ability of proteases from sprouted wheat, rye, and barley to degrade gliadin peptides toxic to celiac patients and their characterization have been described in a study [17]. However, it turned out that not all of them showed activity under acidic conditions. For example, the best-known and currently clinically tested endopeptidase EP-B2 from barley *Hordeum vulgare* has an optimal pH of 7.0 [34]. On the other hand, a pool of proteases from germinating wheat grains, naturally designed to fully digest wheat storage proteins, has been shown to reduce the toxic effects of gluten in vitro and ex vivo [16].

In this study, we analyzed the ability of computationally selected wheat proteases Ta-P7 and Ta-V6, which exhibit predicted high specificity for gluten-specific motifs, in gluten degradation assays and their potential to reduce the toxic effects of the resulting peptides under conditions limited to the gastrointestinal tract compared to Triticain-α. Our early work on Triticain-α showed that this cysteine endopeptidase, unlike the aforementioned plant-derived glutenases [17,34], hydrolyzed the major components of gluten (α-, γ-, ω-gliadins and glutenin) at pH 3–6.5 and 37 °C, making it a potential candidate for gluten degradation as an oral therapy after appropriate in vivo experiments [23].

The recombinant proenzymes Ta-P7 and Ta-V6 have been shown to be produced by expression in bacterial cells and subsequent purification by affinity chromatography due to the polyhistidine sequence introduced into their sequences, which is convenient for potential large-scale protein production. Mature (autoprocessed) forms of Ta-P7 and Triticain-α are detectable over a wide pH range (2.6–7.5) and are not stable at pH 2.6, with activation occurring within 2–5 min under acidic conditions (pH 3.6–4.6) at body temperature (Figure 2A,B), which is the optimal environment for digestion in the stomach, where acidity is thought to vary between 3–6 during ingestion [35]. However, in the case of Ta-V6, we were only able to detect the mature form in a polyacrylamide gel after 8 h of incubation at pH 4.6 (Figure 2C), which is apparently long enough for the glutenase activation process to occur in the gastrointestinal tract. Therefore, the conditions of pH 4.6 at 37 °C were selected for further experiments as the most suitable conditions for the autocatalytic activation of the tested proteases. To confirm that recombinant Ta-P7 and Ta-V6, like Triticain-α, are functionally active proteases in the gastrointestinal environment, we tested their ability to proteolyze gluten and its derived components. By calculating kinetic parameters, we showed that the activated Ta-P7 and Ta-V6, as well as Triticain-α, were able to hydrolyze the model Triticain-α’s substrate (α-gliadin-derived epitope) in the pH range of 3.6–7.5. It was found that the highest affinity to the Ac-PLVQ-AMC was observed for Triticain-α and Ta-V6 at pH 3.6, and for Ta-P7 at a higher value, pH 5.6 (Table 1). On the other hand, the degree of peptide hydrolysis peaked at pH 4.6–5.6 for both Ta-P7 and Triticain-α but was two orders of magnitude higher for the latter. In the case of Ta-V6, the maximum activity was observed at higher pH (5.6–7.5) but also did not reach the corresponding values of Triticain-α (Figure 3). Consequently, according to the kinetic characteristics, the most efficient hydrolysis of the peptide substrate among the studied enzymes under the conditions of the gastrointestinal tract is carried out by Triticain-α. The wheat gluten degradation experiment at pH 4.6 and 37 °C showed that this multi-component protein mixture is almost completely cleaved by both Ta-P7 and Triticain-α within 5 min into low molecular weight products that are not fixed in a 12% polyacrylamide gel. However, it takes longer for Ta-V6 to achieve this effect (Figure 4). To verify that the tested proteases cleave gluten with the formation of non-toxic gliadin degradation products, the glutenase activities of Triticain-α, Ta-P7, and Ta-V6 were measured in the presence of pepsin at pH 4.6 (simulating gastric digestion) and then trypsin at pH 8.0 (simulating intestinal digestion) at 37 °C by enzyme-linked immunosorbent assay. The results clearly showed that Triticain-α and Ta-P7 significantly reduced toxic peptide levels compared to both intact gluten and gluten treated with pepsin-trypsin digestion (Figure 5). In particular, Triticain-α significantly contributes to the reduction of toxic cleavage products of gliadin treated with gastrointestinal enzymes (pepsin and trypsin). Unfortunately, the cleavage of toxic gliadin peptides by Ta-V6 could not be reliably demonstrated in this case.

It should be emphasized that our predictions in the computational screening were based on the AlpaFold statistical model, which considers contacts rather than interaction efficiency. It has been shown previously that for point contacts, there is no correlation between prediction quality and the experimental relative change in Gibbs energy with substrate binding values [36]. In our case of experimental evaluation of selected proteases, the situation was complicated by the wide pH range of the enzyme work, which can strongly affect the interaction between molecules.

To summarize the results obtained, among the wheat proteases computationally selected here, Ta-P7 and Ta-V6, the recombinant Ta-P7 experimentally demonstrated its better glutenase activity at pH 4.6 at 37 °C. However, recombinant Triticain-α, which has already been partially characterized, best meets the in vitro requirements for use in enzyme therapy. We propose that this enzyme, after in vivo experiments, will demonstrate its potential as a promising candidate for the development of an oral drug or dietary supplement for the modification of GFD in celiac patients or individuals suffering from gluten-related disorders.

## 4. Materials and Methods

### 4.1. Computational Screening

In our previous study [22], 181 wheat C1A peptidase sequences were detected. For this study, we used these data and removed 59 sequences with predicted C1A domains of less than 150 amino acids. Then, for each putative enzyme complex with the characterized gluten peptides PLVQLPYP, PQPQLPYP, or VLPQLPYP, AlphaFold2-multimer-v3 [37] implemented in ColabFold v. 1.5.5 was used to predict the structure of the enzyme-substrate complex. Multiple sequence alignment was performed in ColabFold using MMSeqs2. Ten replicates and eight seeds were used for all models. Built-in energy minimization was performed in Amber, resulting in five models for each seed. For each resulting structure from 14,640 models, the distances from the Sγ atom of the cysteine and C in the peptide cleavage site were calculated using the MDTraj Python module [38], and the minimum value was selected for analysis.

### 4.2. Construction of Escherichia coli Expression Vectors

Total plant RNA was extracted from leaves of 2–3-day-old wheat seedlings using the ExtractRNA kit (Evrogen, Moscow, Russia) according to the manufacturer’s specifications. Molecular cloning of the novel wheat glutenases Ta-P7 and Ta-V6 was performed using standard methods similar to the cloning and bacterial expression of Triticain-α [17,20]. Briefly, Ta-P7 and Ta-V6 cDNAs were generated by reverse transcription using specific primers Ta-P7-3′UTR or Ta-V6-3′UTR (listed in Table S2) to obtain oligonucleotides complementary to the 3′ UTR of the respective proteases. The resulting cDNAs were amplified with the specific primers Ta-P7-F and Ta-P7-R or Ta-V6-F and Ta-V6-R (Table S2) and cloned into the pET28a expression vector using *Nhe*I and *Eco*RI restriction sites (Thermo Fisher Scientific Inc., Waltham, MA, USA) to construct expression vectors for the production of N-terminally 6xHis-tagged Ta-P7 or Ta-V6. The fidelity of all constructs described was verified by DNA sequencing.

### 4.3. Protein Expression and Purification

The recombinant plasmids pET28a/Ta-P7 and pET28a/Ta-V6 were separately transformed into *E. coli* Rosetta (DE3) (Novagen, Madison, WI, USA) and induced with IPTG (Novagen, CA, USA) to express N-terminal 6xHis-tagged proteases lacking the N-terminal signal peptide. Bacterially expressed recombinant Ta-P7 and Ta-V6, as well as Triticain-α (lacking the signal peptide and the granulin domain), were produced in soluble form according to our previously described method [20], followed by metal chelate affinity protein isolation and subsequent size exclusion chromatography. To determine the purity and molecular weight of the isolated proenzymes, SDS-PAGE was performed as described by Laemmli [39] using a 14% polyacrylamide resolving gel. Protein concentration was measured at A280 nm using a NanoDrop spectrophotometer (Thermo Scientific, Wilmington, DE, USA).

### 4.4. Autocatalytic Activation

Enzymatically inactive proforms of Ta-P7, Ta-V6, and Triticain-α (as a comparative control) were incubated in different activation buffers consisting of 0.2 M of either glycine, or sodium acetate, or phosphate with pH values from 2.6 to 7.5 at 37 °C [23]. The extent of protease autoprocessing was continuously assessed by SDS-PAGE of the control samples.

### 4.5. Protease Activity Assays

#### 4.5.1. Protease Activity Assays with Fluorogenic Substrate

The activity of preactivated Ta-P7, Ta-V6, and Triticain-α (as a comparative control) was assayed at 37 °C using 20 nM of each enzyme and a concentration range of 5–250 μM of the fluorogenic peptide substrate acetyl-Pro-Leu-Val-Gln-7-amino-4-methylcoumarin (Ac-PLVQ-AMC) (Peptech, Saint Petersburg, Russia) [40] in 0.2 M buffer (pH 3.6–7.5), 100 mM NaCl, 15 mM dithiothreitol, 0.6 mM EDTA, and 0.5% DMSO. The AMC fluorescence (relative fluorescence units, RFU) released over time was assessed at an excitation wavelength of 360 nm and an emission wavelength of 460 nm using a CLARIOstar^®^ fluorescence spectrophotometer (BMG Labtech, Ortenberg, Germany). Reaction rates were determined by linear regression of the initial slope of the progression curves. RFU was converted to the amount of substrate hydrolyzed using a standard curve generated from the fluorescence measurements of the defined AMC concentrations. All enzymatic reactions were performed in triplicate.

#### 4.5.2. Gluten Cleavage

Bulk wheat gluten fraction, isolated from wheat seeds as described [19], was dissolved in 0.2 M glycine buffer (pH 2.6) to a concentration of 4 mg/mL; then, activated enzymes (Ta-P7, Ta-V6 and Triticain-α) were added to it separately in a 20:1 weight ratio [17]. The suspensions were incubated at 37 °C in 0.2 M sodium acetate buffer, pH 4.6, for 1 h with constant vigorous stirring, and then the control aliquots were analyzed on 12% SDS-PAGE in order to detect the resulting protein bands.

### 4.6. ELISA-Based Gluten Toxicity Test

Wheat gluten (50 mg/mL suspension in 0.2 M glycine buffer, pH 2.6) separately mixed with preactivated Triticain-α, Ta-P7, Ta-V6 in a 20:1 weight ratio was incubated with (or without) 50 mg/mL pepsin for 4 h at 37 °C, pH 4.6, and further incubated with 50 mg/mL trypsin for 2 h at pH 8.0. These samples were added to gliadin-coated microtiter wells of the Ridascreen^®^ Gliadin Competition Kit (R7021, R-Biopharm, Darmstadt, Germany) and further manipulations were performed according to the manufacturer’s instructions. The gliadin content of the samples was calculated using the RIDA^®^SOFT Win software (https://food.r-biopharm.com/products/ridasoft-win-net/, accessed on 17 March 2025) available for RIDASCREEN immunoassays. The experiment was performed in triplicate.

### 4.7. Statistical Analysis

All experimental analyses were performed in triplicate, and data are expressed as mean ± SEM. Data were analyzed using GraphPad Prism software version 8.0 (unless otherwise noted). A *p*-value of <0.05 was considered statistically significant.

## Figures and Tables

**Table 1:** Kinetic parameters of Ac-PLVQ-AMC hydrolysis by (Ttc-α), Ta-P7, and Ta-V6 at different pH values at 37 °C. All data are expressed as mean ± SEM; ns—*p* > 0.05; ***—*p* = 0.0001; ****—*p* < 0.0001 vs. kinetic constants of Ttc-α as a comparative control.

Proteases	Kinetic Constants ^§^	pH 3.6	pH 4.6	pH 5.6	pH 6.5	pH 7.5
Ttc-α	*K*_M_, µM	21.5 ± 1.8	34.6 ± 3.1	46.0 ± 3.4	49.4 ± 4.2	582.7 ± 259.9
	*k*_cat_, s^−1^	2.45 ± 0.06	8.48 ± 0.27	9.67 ± 0.29	7.85 ± 0.27	1.01 ± 0.35
Ta-P7	*K*_M_, µM	44.7 ± 6.6 ^ns^	32.2 ± 3.0 ^ns^	24.5 ± 1.9 ^ns^	31.4 ± 2.6 ^ns^	35.3 ± 7.7 ***
	*k*_cat_, s^−1^	0.12 ± 0.01 ****	0.30 ± 0.01 ****	0.24 ± 0.01 ****	0.16 ± 0.00 ****	0.12 ± 0.01 ***
Ta-V6	*K*_M_, µM	16.4 ± 7.3 ^ns^	20.5 ± 1.7 ^ns^	22.6 ± 1.8 ^ns^	26.9 ± 1.8 ^ns^	29.2 ± 3.7 ***
	*k*_cat_, s^−1^	0.07 ± 0.01 ****	1.44 ± 0.04 ****	2.42 ± 0.07 ****	2.64 ± 0.06 ****	2.42 ± 0.11 ****

**^§^**—*K*_M_, Michaelis constant (micromoles of substrate per liter or µM); *k*_cat_, catalytic constant (turnover number of an enzyme per second or s^−1^).

## Data Availability

The original contributions presented in this study are included in the article/Supplementary Material. Further inquiries can be directed to the corresponding author.

## Supplementary Materials

The following supporting information can be downloaded at: https://www.mdpi.com/article/10.3390/ph18040592/s1, Table S1: Minimum and average distances in enzyme-substrate complex models based on AlphaFold2 prediction for all considered proteins and the three substrates PLVQLPYP, PQPQLPYP, and VLPQLPYP, respectively. The last column shows the average value of the minimum distance for the three substrates. The results were sorted according to the last column; Table S2: Primers used for cloning the wheat glutenases Ta-P7 and Ta-V6 (restriction sites are underlined).

## Abbreviations

The following abbreviations are used in this manuscript:

AFm	AlphaFold2-multimer
CD	celiac disease
Glt	gluten
Glt-ase	glutenase
GFD	gluten-free diet
PLCP	papain-like cysteine protease
PT	pepsin and trypsin
Ta-P7	papain-like cysteine protease, Uniprot identifier: A0A3B6JDP7
Ta-V6	papain-like cysteine protease, Uniprot identifier: V6A0A3B6B7V6
Ttc-α	papain-like cysteine protease Triticain-α
UTR	untranslated region
