# Supplementary material for: Computational Screening and Experimental Evaluation of Wheat Proteases for Use in the Enzymatic Therapy of Gluten-Related Disorders
Source: Pharmaceuticals (Basel). 2025 Apr 18;18(4):592. doi: 10.3390/ph18040592 (PMC12030614; doi:10.3390/ph18040592)
Supplement: Supplementary file 1 [file pharmaceuticals-18-00592-s001.zip › Suppl. TableS1.pdf]

Table S1. Minimum and average distances in enzyme-substrate complex models based on AlphaFold prediction for all considered proteins and the three substrates PLVQLPYP, PQPQLPYP, and VLPQLPYP, respectively. The last column shows the average value of the minimum distance for the three substrates. The results were sorted according to the last column.

min-PLVQ	mean-PLVQ	min-PQPQ	mean-PQPQ	min-VLPQ	mean-VLPQ	id	allmean	
3,98	4,00	3,49	3,73	4,10	4,19	TRIAE_AA1165320.1	3,98	Ta-P7
3,87	3,93	3,50	3,59	4,08	4,58	TRIAE_AA0984170.1	4,03	
3,81	4,27	4,04	4,04	3,90	3,91	TRIAE_AA0338690.1	4,08	
3,68	3,88	4,13	4,42	3,41	3,95	TRIAE_AA0095050.1	4,08	
4,00	4,48	3,78	4,25	3,68	3,70	TRIAE_AA1688600.1	4,14	
4,12	4,13	3,37	3,51	3,67	5,04	TRIAE_AA1735060.1	4,23	
4,12	4,16	3,82	4,17	4,32	4,41	TRIAE_AA1296100.1	4,25	
4,06	4,07	4,27	4,39	4,28	4,33	TRIAE_AA1190030.1	4,26	
4,29	4,34	4,54	4,58	3,51	3,92	TRIAE_AA2131470.1	4,28	
4,05	4,13	3,75	4,16	4,46	4,57	TRIAE_AA0530740.1	4,29	
3,85	3,86	3,91	3,92	5,07	5,16	TRIAE_AA0350270.1	4,31	
3,81	3,82	3,87	3,89	5,16	5,26	TRIAE_AA0579930.1	4,32	
4,07	4,49	3,57	3,72	3,56	4,79	TRIAE_AA1353100.1	4,33	
4,05	4,52	3,72	4,82	3,66	3,71	TRIAE_AA0488590.1	4,35	
3,83	3,84	3,16	3,61	5,56	5,63	TRIAE_AA0287470.1	4,36	
3,46	3,83	4,45	4,51	3,99	4,80	TRIAE_AA0248030.1	4,38	
4,20	4,30	4,19	4,62	4,27	4,33	TRIAE_AA0904830.1	4,42	
4,31	4,45	4,29	4,51	3,75	4,42	TRIAE_AA1656610.1	4,46	
3,98	4,47	3,80	4,01	3,71	4,90	TRIAE_AA1961090.1	4,46	
3,82	3,84	3,82	3,84	5,29	5,70	TRIAE_AA0562110.1	4,46	
4,29	4,41	3,58	3,86	5,05	5,13	TRIAE_AA2085470.1	4,47	
3,57	5,04	4,10	3,88	5,74	6,05	TRIAE_AA0280660.1	4,47	Ta-V6
4,14	4,56	3,71	3,94	4,47	4,92	TRIAE_AA1571230.1	4,47	

4,13	4,32	3,98	5,44	3,43	3,81	TRIAE_AA2085540.1	4,52	
4,95	5,02	4,20	4,21	4,25	4,42	TRIAE_AA1708220.1	4,55	
5,00	5,12	4,18	4,26	3,86	4,28	TRIAE_AA1906230.1	4,55	
4,36	4,44	4,76	4,93	3,09	4,29	TRIAE_AA1733400.1	4,55	
5,08	5,14	3,31	3,67	3,94	4,90	TRIAE_AA1341070.1	4,57	
3,76	3,80	4,00	4,00	5,90	5,92	TRIAE_AA1414200.1	4,57	
4,00	4,30	4,36	4,55	4,59	4,90	TRIAE_AA1574170.1	4,58	
4,44	4,51	4,31	4,52	4,37	4,74	TRIAE_AA0753160.1	4,59	
4,53	4,54	4,30	4,81	4,08	4,43	TRIAE_AA1092330.1	4,60	
3,82	4,97	4,45	4,54	4,30	4,38	TRIAE_AA0077320.1	4,63	
4,02	4,56	3,72	3,75	5,57	5,59	TRIAE_AA1247530.2	4,63	
3,95	3,96	3,80	3,82	6,08	6,11	TRIAE_AA0080230.1	4,63	
4,74	4,84	4,22	4,23	4,39	4,89	TRIAE_AA1510630.1	4,66	
3,96	4,50	4,05	4,07	5,27	5,41	TRIAE_AA0514720.1	4,66	
5,11	5,16	4,05	4,15	3,65	4,76	TRIAE_AA0992160.1	4,69	
4,93	4,98	4,32	4,37	3,74	4,81	TRIAE_AA0987260.1	4,72	
4,53	4,66	4,13	4,48	4,45	5,06	TRIAE_AA0584600.1	4,73	
4,09	4,70	4,39	4,45	4,93	5,06	TRIAE_AA0106160.1	4,74	
4,92	4,99	3,96	4,00	4,92	5,24	TRIAE_AA0409930.1	4,74	
3,96	3,96	3,86	3,99	6,35	6,36	TRIAE_AA0430650.2	4,77	
5,00	5,00	3,91	3,94	5,17	5,37	TRIAE_AA1808970.1	4,77	
4,51	4,60	3,61	3,97	5,38	5,74	TRIAE_AA0287890.1	4,77	
3,86	5,10	3,98	4,97	4,18	4,28	TRIAE_AA0170520.1	4,78	
4,07	4,14	3,85	3,87	6,28	6,33	TRIAE_AA0792320.1	4,78	
3,68	5,17	4,15	4,86	3,96	4,37	TRIAE_AA0665220.1	4,80	
4,98	4,99	3,24	3,29	6,08	6,13	TRIAE_AA1688610.1	4,80	
**3,96**	**3,98**	**3,89**	**4,01**	**6,36**	**6,45**	**TRIAE_AA0430650.1**	**4,81**	**Ttc-α**
4,47	4,68	4,43	4,49	4,84	5,34	TRIAE_AA1670770.1	4,84	
5,01	5,15	4,82	4,93	3,52	4,45	TRIAE_AA1674490.1	4,84	
3,82	3,84	3,91	4,06	6,63	6,66	TRIAE_AA0851230.1	4,86	

3,98	4,05	3,21	5,20	5,30	5,46	TRIAE_AA0198300.2	4,90
3,96	4,59	3,50	3,81	6,36	6,46	TRIAE_AA1889150.1	4,95
3,53	3,95	5,42	5,62	5,11	5,35	TRIAE_AA0028760.1	4,97
3,83	3,85	3,80	4,40	6,68	6,69	TRIAE_AA0661280.1	4,98
4,91	4,96	3,73	3,82	6,17	6,21	TRIAE_AA1503810.1	5,00
4,75	4,88	4,95	4,99	5,05	5,18	TRIAE_AA1491460.1	5,02
4,93	4,95	3,86	3,98	6,15	6,16	TRIAE_AA0384430.1	5,03
4,06	4,64	3,92	3,94	6,51	6,53	TRIAE_AA0698060.1	5,04
4,17	4,17	4,47	5,80	4,31	5,22	TRIAE_AA0736470.1	5,06
5,15	5,15	3,85	4,22	6,13	6,20	TRIAE_AA0851210.1	5,19
4,39	4,40	4,49	4,84	6,23	6,44	TRIAE_AA1544330.1	5,22
5,01	5,02	4,42	5,26	5,08	5,56	TRIAE_AA1447130.1	5,28
5,69	6,04	3,98	4,95	4,09	4,93	TRIAE_AA0417630.1	5,31
4,03	5,08	4,69	4,76	6,06	6,11	TRIAE_AA0963810.1	5,32
4,87	4,90	5,83	5,94	5,02	5,48	TRIAE_AA1954290.1	5,44
4,15	5,00	4,12	4,43	6,89	6,89	TRIAE_AA1238050.1	5,44
4,90	4,96	6,21	6,31	4,44	5,07	TRIAE_AA1596190.1	5,45
5,34	5,37	4,90	4,98	5,91	5,99	TRIAE_AA1905950.1	5,45
4,38	4,43	6,95	7,10	4,48	4,88	TRIAE_AA1361060.1	5,47
4,10	4,12	5,61	5,94	6,33	6,36	TRIAE_AA0881370.1	5,48
4,09	4,24	6,53	6,53	5,22	5,89	TRIAE_AA1251030.3	5,55
4,14	4,28	6,54	6,54	5,23	5,90	TRIAE_AA1251030.1	5,57
3,97	4,26	6,67	6,68	5,97	6,06	TRIAE_AA0846860.1	5,67
4,89	4,93	6,78	6,85	3,35	5,39	TRIAE_AA0569140.1	5,72
5,65	5,66	3,92	5,22	6,22	6,30	TRIAE_AA1860790.1	5,73
4,46	10,03	3,46	3,62	3,36	3,58	TRIAE_AA1094100.2	5,74
4,23	4,65	6,99	7,00	5,45	5,60	TRIAE_AA0126990.1	5,75
4,19	4,26	6,70	6,73	6,00	6,32	TRIAE_AA0433440.1	5,77
3,86	4,09	5,57	5,89	6,09	7,38	TRIAE_AA1174650.2	5,79
4,97	5,50	4,74	6,25	5,60	5,72	TRIAE_AA0320600.1	5,82

4,55	4,75	4,93	7,96	4,95	5,01	TRIAE_AA0821120.1	5,91
4,21	9,48	3,41	3,77	3,60	5,09	TRIAE_AA1183770.1	6,11
6,69	6,70	3,37	4,77	6,85	7,53	TRIAE_AA1860880.1	6,33
6,37	7,85	5,80	6,41	4,56	4,88	TRIAE_AA0328120.1	6,38
6,02	6,31	6,06	6,60	6,56	6,97	TRIAE_AA1796320.1	6,63
6,87	6,87	6,82	6,84	6,62	6,74	TRIAE_AA0318200.1	6,81
6,88	6,93	7,73	7,91	4,13	5,96	TRIAE_AA1860860.2	6,94
4,92	5,00	10,54	10,65	5,09	5,25	TRIAE_AA1277960.1	6,97
7,06	7,13	7,13	7,14	6,83	6,87	TRIAE_AA1833980.1	7,05
4,64	4,73	10,80	10,90	5,46	5,63	TRIAE_AA0817310.1	7,09
9,22	9,54	7,29	7,65	7,00	7,46	TRIAE_AA0429310.1	8,22
8,74	8,96	8,84	9,45	7,36	7,65	TRIAE_AA0093620.1	8,69
8,52	8,55	8,56	9,39	8,43	8,64	TRIAE_AA0664490.1	8,86
7,01	7,81	8,76	8,81	9,24	10,03	TRIAE_AA1389260.2	8,88
9,13	10,57	6,48	7,42	8,85	9,03	TRIAE_AA1785720.1	9,01
14,75	14,92	4,74	5,08	4,97	8,29	TRIAE_AA1994620.1	9,43
4,36	4,45	3,39	3,82	21,30	21,49	TRIAE_AA0850000.1	9,92
14,73	14,78	6,39	6,42	5,56	13,13	TRIAE_AA0322380.2	11,44
15,85	16,11	4,18	9,15	4,43	9,23	TRIAE_AA1062300.1	11,50
14,47	14,50	4,38	4,78	14,46	17,42	TRIAE_AA1994190.1	12,23
5,01	13,09	19,27	19,46	3,85	4,20	TRIAE_AA0565030.1	12,25
9,58	11,31	4,41	4,44	21,74	21,75	TRIAE_AA1730630.1	12,50
14,82	15,34	14,06	14,38	7,95	8,07	TRIAE_AA0967610.1	12,60
14,35	14,36	3,87	4,49	20,38	20,40	TRIAE_AA1777530.1	13,08
9,01	13,32	11,32	11,44	11,21	14,56	TRIAE_AA0577990.1	13,11
15,49	15,49	3,46	3,53	20,49	20,51	TRIAE_AA0312960.1	13,18
12,57	12,73	3,89	8,36	20,46	20,47	TRIAE_AA1737060.1	13,85
16,94	16,96	5,82	5,83	20,90	20,96	TRIAE_AA0443250.1	14,58
17,10	17,15	6,07	6,15	20,66	20,80	TRIAE_AA0528870.1	14,70
16,86	16,96	13,09	13,51	13,18	13,82	TRIAE_AA1316240.1	14,76

13,84	13,94	3,75	9,07	21,36	21,41	TRIAE_AA1053890.1	14,81
17,75	17,77	6,99	7,17	20,43	20,47	TRIAE_AA1189610.2	15,14
15,48	15,49	15,05	15,10	15,13	15,26	TRIAE_AA0523110.1	15,28
12,58	13,82	14,31	14,32	14,34	18,39	TRIAE_AA0559420.1	15,51
15,44	15,54	16,19	16,22	16,12	16,16	TRIAE_AA0491420.1	15,97
16,79	16,86	16,51	19,63	14,19	14,36	TRIAE_AA0986230.1	16,95
13,52	16,30	14,03	14,53	20,22	20,24	TRIAE_AA2006450.1	17,02
16,82	17,30	16,55	17,08	17,32	17,66	TRIAE_AA0516850.1	17,35
14,16	14,68	22,14	22,14	14,38	17,76	TRIAE_AA0425030.1	18,19
1 000,00	1 000,00	1 000,00	1 000,00	1 000,00	1 000,00	TRIAE_AA0198310.1	1 000,00
1 000,00	1 000,00	1 000,00	1 000,00	1 000,00	1 000,00	TRIAE_AA1325870.3	1 000,00
1 000,00	1 000,00	1 000,00	1 000,00	1 000,00	1 000,00	TRIAE_AA0555000.1	1 000,00
1 000,00	1 000,00	1 000,00	1 000,00	1 000,00	1 000,00	TRIAE_AA0712240.1	1 000,00
1 000,00	1 000,00	1 000,00	1 000,00	1 000,00	1 000,00	TRIAE_AA0226230.2	1 000,00
1 000,00	1 000,00	1 000,00	1 000,00	1 000,00	1 000,00	TRIAE_AA0901580.1	1 000,00
1 000,00	1 000,00	1 000,00	1 000,00	1 000,00	1 000,00	TRIAE_AA1000830.1	1 000,00
1 000,00	1 000,00	1 000,00	1 000,00	1 000,00	1 000,00	TRIAE_AA2139910.1	1 000,00
1 000,00	1 000,00	1 000,00	1 000,00	1 000,00	1 000,00	TRIAE_AA0431940.1	1 000,00
1 000,00	1 000,00	1 000,00	1 000,00	1 000,00	1 000,00	TRIAE_AA1274070.1	1 000,00
1 000,00	1 000,00	1 000,00	1 000,00	1 000,00	1 000,00	TRIAE_AA0332310.1	1 000,00
1 000,00	1 000,00	1 000,00	1 000,00	1 000,00	1 000,00	TRIAE_AA1157190.1	1 000,00
1 000,00	1 000,00	1 000,00	1 000,00	1 000,00	1 000,00	TRIAE_AA0326660.1	1 000,00
1 000,00	1 000,00	1 000,00	1 000,00	1 000,00	1 000,00	TRIAE_AA1672530.1	1 000,00
1 000,00	1 000,00	1 000,00	1 000,00	1 000,00	1 000,00	TRIAE_AA1298890.1	1 000,00
1 000,00	1 000,00	1 000,00	1 000,00	1 000,00	1 000,00	TRIAE_AA0170120.1	1 000,00
1 000,00	1 000,00	1 000,00	1 000,00	1 000,00	1 000,00	TRIAE_AA1163270.1	1 000,00
1 000,00	1 000,00	1 000,00	1 000,00	1 000,00	1 000,00	TRIAE_AA0106170.1	1 000,00
1 000,00	1 000,00	1 000,00	1 000,00	1 000,00	1 000,00	TRIAE_AA0626080.1	1 000,00
1 000,00	1 000,00	1 000,00	1 000,00	1 000,00	1 000,00	TRIAE_AA0723140.1	1 000,00
1 000,00	1 000,00	1 000,00	1 000,00	1 000,00	1 000,00	TRIAE_AA2172050.1	1 000,00

1 000,00	1 000,00	1 000,00	1 000,00	1 000,00	1 000,00	TRIAE_AA2157820.1	1 000,00	
