# Supplementary material for: Computational Screening and Experimental Evaluation of Wheat Proteases for Use in the Enzymatic Therapy of Gluten-Related Disorders
Source: Pharmaceuticals (Basel). 2025 Apr 18;18(4):592. doi: 10.3390/ph18040592 (PMC12030614; doi:10.3390/ph18040592)
Supplement: Supplementary file 1 [file pharmaceuticals-18-00592-s001.zip › Suppl. TableS2.pdf]

Table S2. Primers used for cloning the wheat glutenases Ta-P7 and Ta-V6 (restriction sites are underlined).

Primer	Sequence 5’-3’
Ta-P7-3’UTR	TGATCATATGTGTGCA
Ta-P7-F	CTAG<u>GCTAGC</u>ATCCCGTTCACGGAG
Ta-P7-R	ACGC<u>GAATTC</u>CTAGAGCTCATCACTATGAG
Ta-V6-3’UTR	GAATCAACAAGATCTGAGACCG
Ta-V6-F	TATA<u>GCTAGC</u>CACGTCGACGACGCCG
Ta-V6-R	TACG<u>GAATTC</u>CTAGCAGCATCCCCACGC
